# Multicenter Phase 2 Study about the Safety of No Antimicrobial Prophylaxis Use in Low-Risk Patients Undergoing Laparoscopic Distal Gastrectomy for Gastric Carcinoma (KSWEET-01 Study)

**DOI:** 10.1155/2017/8928353

**Published:** 2017-06-05

**Authors:** Oh Jeong, Mi Ran Jung, Seong Yeob Ryu, Young-Kyu Park, Min Chan Kim, Ki Han Kim, Seung Wan Ryu, In Gyu Kwon, Young Gil Son

**Affiliations:** ^1^Department of Surgery, Chonnam National University School of Medicine, Gwangju, Republic of Korea; ^2^Department of Surgery, Dong-A University School of Medicine, Busan, Republic of Korea; ^3^Department of Surgery, Keimyung University School of Medicine, Daegu, Republic of Korea

## Abstract

**Background:**

Recent studies have shown a lower risk of surgical site infections (SSI) after laparoscopic distal gastrectomy compared to open surgery. This is a phase 2 study aiming to determine the incidence of SSI after laparoscopic distal gastrectomy without using antimicrobial prophylaxis (AMP).

**Methods:**

cT1N0 gastric cancers that were subject to laparoscopic distal gastrectomy were enrolled. Based on the unacceptable SSI incidence of ≥12.5% and the target SSI incidence of ≤5%, 105 patients were enrolled with an *α* of 0.05 and a power of 80% (ClinicalTrials.gov, NCT02200315).

**Results:**

In intention-to-treat analysis, patients did not reach the target SSI rate (12.4%, 95% confidence  interval = 6.8%–19.8%). Of patients, 44 patients had a protocol violation, such as extended lymph node dissection (LND) or inappropriate nonpharmacological SSI prevention measures. Per-protocol analysis excluding these patients (*n* = 61) showed a SSI rate of 4.9%, which was within the target SSI range. Multivariate analysis revealed that extracorporeal anastomosis and extended LND were independent risk factors for SSI.

**Conclusions:**

This study failed to reach the target SSI rate without using AMP. However, per-protocol analysis suggests that no AMP might be feasible when limited LND and adequate SSI prevention measures were performed.

## 1. Introduction

Since laparoscopic distal gastrectomy was first reported in 1994 [1], it has been widely adopted for the treatment of early-stage gastric cancer in Korea, China, and Japan [2]. Since laparoscopic surgery is associated with smaller surgical wounds and minimal tissue damage, it could reduce the risk of postoperative complications in patients undergoing laparoscopic distal gastrectomy. For example, a comprehensive meta-analysis of six randomized controlled trials and 19 nonrandomized studies comparing open and laparoscopic distal gastrectomy showed that the laparoscopic surgery group had lower overall complications, medical complications, and minor surgical complications than the open surgery group [3]. A recent large multicenter randomized controlled trial in Korea (KLASS-01) has also demonstrated that complication rates were significantly lower after laparoscopic distal gastrectomy than those after open distal gastrectomy, particularly in the incidences of wound complications [4].

Antimicrobial prophylaxis (AMP) for various clean or clean-contaminated surgical procedures has long been the standard to prevent postoperative surgical site infections (SSI). Compared to open gastrectomy, recent studies on laparoscopic gastrectomy have shown significantly lower incidences of SSI ranging from 2% to 4% [4–6]. Current guidelines recommend the single intraoperative use of cefazoline for gastric cancer surgery [7]. However, previous studies on the use of AMP for gastric cancer surgery have mostly focused on patients undergoing open gastrectomy [8, 9].

Reducing the use of antibiotics could help reduce the medical costs, prevent the antibiotics-related complications and emergence of resistant strains. Recently, several randomized controlled trials have demonstrated that AMP can be safely omitted for low-risk patients undergoing laparoscopic cholecystectomy [10]. With advances in surgical techniques and instruments, the risk of SSI after laparoscopic distal gastrectomy is considered as low as that after laparoscopic cholecystectomy. Therefore, in this preliminary phase 2 trial, we investigated the incidence of SSI without AMP in low-risk patients undergoing laparoscopic distal gastrectomy for gastric carcinoma.

## 2. Methods

### 2.1. Study Design

This was a multicenter single-arm phase 2 study to investigate the incidence of SSI after laparoscopic distal gastrectomy without AMP. Inclusion criteria were histologically proven gastric adenocarcinoma (cT1N0) that were treated with laparoscopic distal gastrectomy and limited (D1+) lymph node dissection (LND), age of patients between 18 and 65 years, European Cooperative Oncology Group (ECOG) performance status of 0 to 1, and adequate bone marrow, hepatic, renal, pulmonary, and cardiac function. Patients were excluded if they had active infections, other major organ resection, preoperative chemotherapy or radiotherapy, poorly controlled diabetes mellitus or hypertension, or malnutrition (preoperative weight loss ≥10% or body mass index of ≤18 kg/m^2^).

The primary endpoint was the incidence of SSI within 30 days postsurgery. SSI was defined based on the diagnostic criteria of the National Nosocomial Infections Surveillance (NNIS) system by Centers for Disease Control and Prevention (CDC) [11]. Secondary outcomes were the incidences of infection at remote sites and postoperative hospital courses including postoperative fever (≥38°C), length of hospital stay, and other complications.

Three institutions that operate more than 200 gastric cancer surgeries per year participated in this study. This study was approved by the institutional review board at each institution. All patients provided written informed consent before entering the study. This study was registered at ClinicalTrials.gov (NCT02200315).

### 2.2. Operative Techniques and Perioperative Care

During surgery, four to five abdominal trocars were used as appropriate. The choice of operative approach (extra- or intracorporeal anastomosis) and reconstruction technique (Billroth I, Billroth II, or Roux-en-Y gastrojejunostomy) were decided as per the surgeon's preference. If extracorporeal anastomosis was performed, a 5 to 6 cm long minilaparotomy was performed at the upper abdomen. In principle, D1+ LND with partial omentectomy was the choice of procedure in the protocol. However, D2 LND and total omentectomy were allowed in cases with more than T2 or N1 disease observed in the operative findings. Abdominal drain and nasogastric tube were selectively used at the surgeon's discretion.

Based on existing evidence [12], we developed the following nonpharmacological SSI prevention measures to reduce the risk of infection in patients without AMP: (1) preoperative body shower or bath using bacteriostatic soap, (2) maintaining intraoperative normothermia, (3) intra-abdominal irrigation with more than 1 L of fluid, and (4) high oxygen supply on the operation day. Other postoperative cares including oral nutrition, pain management, and intravenous fluid administration were followed as per each institution's practice.

### 2.3. Statistics

Prior to this study, we analyzed the data of 1075 patients (not published) and found that three participating institutions showed similar SSI incidences of around 5% after laparoscopic distal gastrectomy. Based on this, we determined a target SSI rate of ≤5% and an unacceptable SSI rate of ≥12.5% (relative risk = 2.5) in patients without using AMP. Based on Simon's two-stage design, 105 patients were needed to test this hypothesis (H0; SSI rate ≥ 12.5%, H1: SSI rate ≤ 5%) with an *α* of 0.05 and a power of 80%. We performed analyses in two groups, namely, intention-to-treat group and per-protocol group. The intention-to-treat group included all patients who were enrolled in the study, whereas the per-protocol group included patients who did not have violations in the operative techniques or SSI prevention measures as outlined in the protocol. All statistical analyses were performed using SPSS version 22.0 (IBM Corp., NY, USA), and two-sided *p* values of <0.05 were considered statistically significant.

## 3. Results

### 3.1. Patient Characteristics

**Figure 1:**
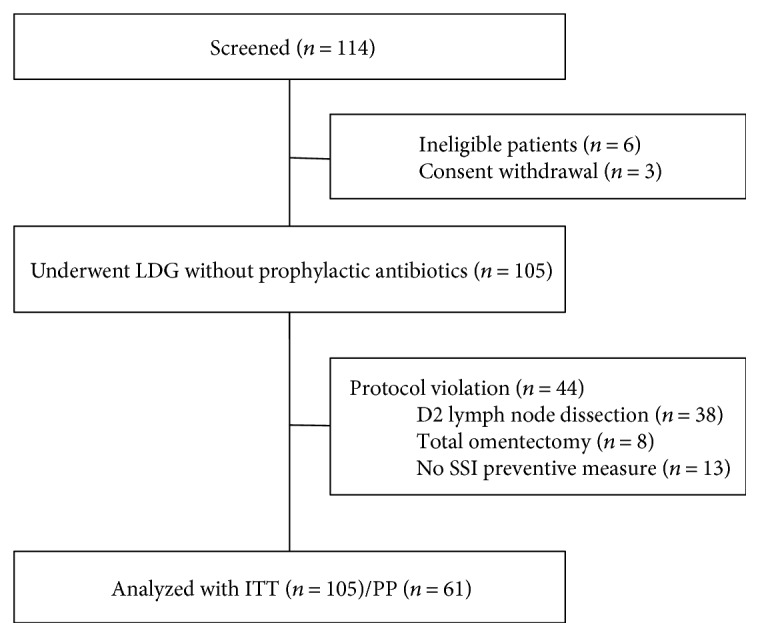
Consort flow diagram. ITT—intention-to-treat group; PP—per-protocol group; SSI—surgical site infection.

Between June 2014 and July 2015, 114 patients were screened and 105 patients were finally enrolled (Figure 1). Demographic and clinicopathological characteristics of patients are summarized in Table 1. Study groups consisted of 60 men and 35 women with a mean age (years) of 51.6. Within these, 41 (39.0%) patients had comorbidities, among which diabetes mellitus and hypertension were the most common. Most patients (*n* = 102) underwent total laparoscopic distal gastrectomy with intracorporeal anastomosis. With respect to reconstruction techniques, Billroth I, Billroth II, and Roux-en-Y gastrojejunostomy were performed in 5, 50, and 50 patients, respectively. In the final pathological examination, there were 99 patients with stage I, nine patients with stage II, and two patients with stage III carcinomas according to the UICC/AJCC TNM classification (7th edition).

### 3.2. Postoperative Outcomes and SSI

In overall patients, the postoperative complication rate was 24.8% (*n* = 26) and there was no hospital mortality (Table 2). The mean hospital stay was 8.7 ± 5.3 days. Thirteen patients (12.4%) developed SSI (95% CI = 6.8%–19.8%) including wound (*n* = 7) and abdominal infections (*n* = 6). These outcomes did not achieve the target SSI rate of ≤5% in this study (Table 2). Among the patients with SSI, two patients with an abdominal infection required radiologic intervention, and others recovered with conservative management.

A total of 38 patients had a protocol violation in the operative techniques (D2 LND or total omentectomy). Furthermore, thirteen patients did not receive adequate SSI prevention measures as outlined in the protocol. Overall, 41 (39.0%) patients experienced a violated protocol in either the operative techniques or SSI prevention measures (Figure 1). However, as shown in Table 3, the incidence of SSI in the per-protocol group (*n* = 61) was 4.9% (95% CI = 0.0%–10.34%), which was within the target SSI rate of this study (H0: SSI rate ≥ 12.5%, H1: SSI rate ≤ 5%, *p* = 0.049). There were 3 cases of wound infections but no abdominal infections occurred in this group.

### 3.3. Risk Factor Analysis for SSI

Table 4 shows univariate and multivariate analyses of risk factors for developing SSI. In the univariate analysis, higher body mass index, lower preoperative albumin levels, extracorporeal anastomosis, D2 LND, and inappropriate SSI preventative measures were associated with an increase in the frequency of SSI (all *p* values ≤ 0.1). Multivariate analysis of these factors revealed that operative factors including extracorporeal anastomosis (odds ratio (OR) = 78.70, 95% confidence interval (CI) = 3.15–1948.98) and D2 LND (OR = 7.45, 95% CI = 1.15–48.06) were independent risk factors for developing SSI.

## 4. Discussion

With the introduction of laparoscopic surgery, the operative quality and surgical outcomes of gastric cancer surgery have significantly improved over the past few decades. Accordingly, the incidences of SSI after laparoscopic gastrectomy have become significantly lower compared to that in the era of open surgery [5, 6]. Given this low risk, this phase 2 trial examined the incidence of SSI without using AMP in low-risk patients undergoing laparoscopic distal gastrectomy. In this study, the intention-to-treat group did not reach the target SSI, which was thought to be attributed to relatively many cases with a protocol violation, such as extended lymph node dissection or inappropriate nonpharmacological SSI prevention measures. However, the per-protocol group that underwent limited LND and adequate SSI prevention measures showed an acceptable SSI without AMP after laparoscopic distal gastrectomy. Our analysis showed that operative techniques, such as extended LND and extracorporeal anastomosis, are important factors increasing the risk of SSI after laparoscopic distal gastrectomy. Unfortunately, our study failed to show the feasibility of no AMP use in patients undergoing laparoscopic distal gastrectomy. However, our study suggests the possibility that nonpharmacological SSI prevention measures may be sufficient to prevent SSI in patients undergoing intracorporeal anastomosis and limited LND during laparoscopic distal gastrectomy. Therefore, we believe that more studies will be warranted to investigate the feasibility of no AMP use in totally laparoscopic distal gastrectomy with limited LND for early gastric cancer in the future.

The overuse of prophylactic antibiotics in gastrointestinal surgery is relatively common. For example, according to a large nationwide survey in Japan, more than 50% of surgeons reported administering of AMP until 3 to 4 days postsurgery in clean-contaminated operations including abdominal surgery [13]. Another study showed that as many as 11 out of 14 high-volume centers in Korea and Japan administered AMP for longer than 24 hours after gastric cancer surgery [14]. Current guidelines recommend a single dose of AMP for gastric cancer surgery and emphasize that appropriate administration within 60 minutes before incision is more efficient than the postoperative extended use of AMP [7]. Some may argue that reducing a single dose of AMP to no use in laparoscopic distal gastrectomy may be trivial for individual patients. However, developing resistance to antibiotics is an alarming level [15]. For instance, in the U.S., more than two million people get infections that are resistant to antibiotics and at least 23,000 people die as a result according to the report issued by Centers for Disease Control and Prevention [16]. Considering this alarming level of increasing resistance of bacteria and associated medical cost, efforts to reduce unnecessary antibiotic use will be essential to improve the quality of surgical care and to prevent emergence of resistant strains.

Studies on the use of AMP for gastric cancer surgery are limited. Recently, two randomized controlled trials have demonstrated that a single use of intraoperative AMP is as effective as extended postoperative use to prevent SSI after gastric cancer surgery [8, 9]. However, these studies are mostly limited to open surgery, and no previous studies have appraised the true efficacy of AMP in laparoscopic gastrectomy. In this preliminary phase 2 trial, the incidence of SSI after laparoscopic distal gastrectomy without AMP was 4.9% in the per-protocol group, which is comparable to previous studies and our historical data. Given that nearly half of all gastric cancer patients are being treated with laparoscopic approaches in our regions, we expect that the use of antibiotics could be significantly reduced if we can omit AMP for laparoscopic gastrectomy.

AMP has long been the standard for preventing SSI. However, preventing SSI does not entirely rely on antibiotics. Preoperative bathing or showering with antiseptic soap is considered a good clinical practice to ensure that the skin is as clean as possible before surgery and reduces the bacterial load, particularly at the site of incision [17]. Recent studies suggest that attention to intraoperative temperature control and supplemental oxygen administration may also decrease the risk of SSI [12]. Theoretically, hypothermia and subsequent tissue hypoxia reduce oxidative killing by neutrophils via reducing the availability of tissue oxygen and impairing the production of reactive oxygen intermediates [18]. Hypothermia also increases the risk of infections by increasing blood loss, thus, requiring blood transfusion [19]. Based on these notions, we applied nonpharmacological prevention measures to reduce SSI in patients without using AMP. As a result, we found that these SSI prevention measures are helpful for reducing the risk of SSI (OR = 0.24, 95% CI = 0.06–0.95). The importance of nonpharmacological measures to prevent SSI should be emphasized in the practice of surgical care.

The incidence of infectious complications after laparoscopic cholecystectomy is extremely low and is significantly lower compared to that after open surgery. A meta-analysis of 15 randomized controlled trials evaluating AMP in elective laparoscopic cholecystectomy showed no significance of AMP in preventing SSI in low-risk patients undergoing laparoscopic cholecystectomy [10]. Given this low risk of SSI, current guidelines do not support routine use of AMP in elective laparoscopic cholecystectomy [7]. Meanwhile, advantages of laparoscopic gastrectomy, such as small incision, minimal tissue damage, and no exposure of abdominal cavity, have also led to significantly lower incidences of SSI (2% to 4%) compared to that in open gastrectomy [4–6]. Theoretically, gastrectomy and cholecystectomy are both classified as a clean-contaminated surgery, which means they have a similar risk of SSI. However, we should be careful to extrapolate the data from laparoscopic cholecystectomy to gastrectomy because of the longer operating time and much blood loss. Optimal AMP use should be guided based on the clinical evidence considering different aspects of each type of surgery. Therefore, we performed this study on the premise that nonpharmacologic prevention measures would be sufficient for preventing SSI in low-risk patients undergoing laparoscopic distal gastrectomy.

There are a few limitations in this study. As we noted earlier, the number of patients with protocol violations was too high due to the lack of experience in early phases of the study. As a result, per-protocol analysis may be underpowered due to a small sample size. Second, this study only included low-risk patients in terms of age, comorbidity, and nutrition status. Besides, participating institutions were highly specialized centers that operate more than 200 cases of gastric cancer per year. Finally, this is a preliminary phase 2 study that examined SSI incidence with historic controls. A randomized controlled trial will be warranted to further investigate the role of AMP in laparoscopic distal gastrectomy.

In conclusion, this study failed to show the target SSI rate in patients undergoing laparoscopic distal gastrectomy for gastric carcinoma. However, per-protocol analysis and risk factor analysis showed the possibility that nonpharmacological SSI prevention measures might be sufficient to prevent SSI when limited lymph node dissection and intracorporeal anastomosis are performed during laparoscopic distal gastrectomy. Therefore, we believe more studies will be warranted to test the feasibility of no AMP use in selected patients undergoing laparoscopic distal gastrectomy for gastric carcinoma.

## Figures and Tables

**Table 1:** Patient characteristics.

	Patients (*n* = 105)
Age (years)	51.6 ± 8.3
Gender	
Male	70 (66.7)
Female	35 (33.3)
BMI (kg/m^2^)	24.7 ± 3.6
ASA status	
1	90 (85.7)
2-3	15 (12.4)
Comorbidity	41 (39.0)
Abdominal operation history	19 (18.1)
Preoperative hemoglobin	
Mean	14.4 ± 1.4
<12 g/dl	5
≥12 g/dl	100
Preoperative albumin	
Mean	4.7 ± 0.4
<3.1 g/dl	0
≥3.1 g/dl	105
Operative approach	
Totally laparoscopic	102 (97.1)
Laparoscopy assisted	3 (2.9)
Reconstruction	
Billroth I	5 (4.8)
Billroth II	50 (47.6)
Roux-en-Y	50 (47.6)
Lymphadenectomy	
D1+	67 (63.9)
D2	38 (36.2)
Omentectomy	
Partial	97 (92.4)
Complete	8 (7.6)
Operating time (min)	213 ± 62
Operative blood loss (ml)	43 ± 34
Harvested lymph nodes	52 ± 18
Tumor location	
Lower	69 (65.7)
Middle	36 (34.3)
Tumor depth	
T1a	61 (58.1)
T1b	36 (34.3)
T2	3 (2.9)
T3	4 (3.8)
T4a	1 (1.0)
Nodal metastasis	
N0	98 (93.3)
N1	6 (5.7)
N3a	1 (1.0)

BMI: body mass index; ASA: American Society of Anesthesiologists score.

**Table 2:** Postoperative outcomes in the overall group.

	Patients (*n* = 105)
Diet start (POD)	2.2 ± 2.7
Gas passage (POD)	2.6 ± 0.8
Postoperative fever	38 (36.2)
Transfusion	1 (1.0)
Hospital stay (POD)	8.7 ± 5.3
Overall complication	26 (24.8)
Wound complication	7
Abdominal infection	6
Gastric stasis	5
Paralytic ileus	4
Anastomosis leak	1
Atelectasis	1
Omental infarction	1
Mortality	0
Surgical site infection	13 (12.4)
Superficial incisional (grade I/II/III/IV)	6 (5/1/0/0)
Deep incisional (grade I/II/III/IV)	1 (0/1/0/0)
Organ/space (grade I/II/III/IV)	6 (0/4/2/0)

Data are expressed as mean ± SD or *n* (%); POD: postoperative day.

**Table 3:** Postoperative outcomes in the per-protocol group.

	Patients (*n* = 61)
Overall complication	13 (21.3)
Surgical site infection	3 (4.9)
Superficial incisional (grade I/II/III/IV)	3 (3/0/0/0)
Deep incisional	0
Organ/space	0
Diet start (POD)	2.0 ± 3.4
Gas passage (POD)	2.3 ± 0.8
Postoperative fever	22 (36.1)
Hospital stay (POD)	8.3 ± 5.0

Data are expressed as mean ± SD or *n* (%); POD: postoperative day.

**Table 4:** Multivariate analysis of risk factors for SSI.

	Univariate		Multivariate	
	OR (95% CI)	*p*	Adjusted OR (95% CI)	*p*
Age (years)	1.04 (0.97–1.13)	0.281		
Male	1.29 (0.39–4.29)	0.676		
BMI	1.16 (0.99–1.35)	0.073	1.19 (0.99–1.45)	0.058
Comorbidity	0.66 (0.19–2.30)	0.516		
Preoperative albumin	0.14 (0.03–0.79)	0.025	0.45 (0.03–7.39)	0.574
Extracorporeal anastomosis	16.55 (1.39–197.70)	0.027	78.70 (3.18–1948.98)	0.008
D2 LND	7.62 (1.95–29.82)	0.004	7.45 (1.15–48.06)	0.035
RYGJ	0.17 (0.03–0.81)	0.026	0.17 (0.02–1.48)	0.108
Omentectomy	1.01 (0.11–8.96)	0.992		
Operating time	0.99 (0.98–1.00)	0.166		
Operative blood loss	1.01 (0.99–1.02)	0.151		
SSI preventive measures	0.24 (0.06–0.95)	0.043	0.63 (0.11–3.79)	0.613

BMI: body mass index; LND: lymph node dissection; RYGJ: Roux-en-Y gastrojejunostomy; SSI: surgical site infection; OR: odds ratio; CI: confidence interval.

## Additional Points

*Key Points*. This phase 2 trial did not reach the target surgical site infection rate without using antimicrobial prophylaxis after laparoscopic distal gastrectomy. However, per-protocol analysis showed that the surgical site infection rate was acceptable without antimicrobial prophylaxis when intracorporeal anastomosis and limited lymph node dissection were performed.
